# Occupational Injuries and Use of Benzodiazepines: A Systematic Review and Metanalysis

**DOI:** 10.3389/fnhum.2021.629719

**Published:** 2021-05-13

**Authors:** Sergio Garbarino, Paola Lanteri, Nicola Luigi Bragazzi, Giovanni Gualerzi, Matteo Riccò

**Affiliations:** ^1^Department of Neuroscience, Rehabilitation, Ophthalmology, Genetics, and Maternal/Child Sciences (DINOGMI), University of Genoa, Genoa, Italy; ^2^UOC Neurophysiopathology, Fondazione IRCCS, Istituto Neurologico “C. Besta,” Milan, Italy; ^3^Laboratory for Industrial and Applied Mathematics, Department of Mathematics and Statistics, York University, Toronto, ON, Canada; ^4^Department of Medicine and Surgery, School of Medicine, University of Parma, Parma, Italy; ^5^AUSL-IRCCS di Reggio Emilia—Department of Public Health, Service for Health and Safety in the Workplace, Reggio Emilia, Italy

**Keywords:** benzodiazepines, side effects, psychotropic drugs, occupational injuries, accidents

## Abstract

**Background:** Benzodiazepines have been widely used in clinical practice for over four decades and continue to be one of the most consumed and highly prescribed class of drugs available in the treatment of anxiety, depression, and insomnia. The literature indicates that Benzodiazepine users at a significantly increased risk of Motor Vehicle accidents compared to non-users but the impact on injuries at workplace is not well-defined. We aimed to investigate whether use of benzodiazepine is associated with increased risk of occupational injuries (OI).

**Methods:** PubMed, Embase, and Scopus databases were searched. A meta-analysis was performed to calculate odds ratio (OR) and 95% confidence interval (CI) among case controls, cross-sectional studies, either questionnaire or laboratory exams based.

**Results:** A total of 13 studies met inclusion criteria, involving 324,168 OI from seven different countries, with an estimated occurrence of benzodiazepine positivity of 2.71% (95% CI 1.45–4.98). A total of 14 estimates were retrieved. Of them, 10 were based on laboratory analyses, three on institutional databases, while one study was based on questionnaires. Regarding the occupational groups, three estimates focused on commercial drivers (0.73%, 95% CI 0.12–4.30), that exhibited a reduced risk ratio for benzodiazepine positivity compared to other occupational groups (RR 0.109, 95% CI 0.063–0.187). Eventually, no increased risk for benzodiazepine positivity was identified, either from case control studies (OR 1.520, 95% CI 0.801–2.885, *I*^2^ 76%), or cross sectional studies, when only laboratory based estimates were taken in account (OR 0.590, 95% CI 0.253–1.377, *I*^2^ 63%).

**Conclusions:** Even though benzodiazepines have the potential to increase injury rates among casual and chronic users, available evidence are insufficient to sustain this hypothesis, particularly when focusing on laboratory-based studies (i.e., studies the characterized the benzodiazepine immediately before the event).

## Introduction

Benzodiazepines (BDZ) are commonly prescribed central nervous system (CNS) drugs that cause a selective allosteric potentiation of the action of γ-aminobutyric acid (GABA) at GABAA receptors, with eventual inhibition of neural activity (Manthey et al., 2014; Bénard-Laribière and Pariente, 2018; Kowalski-McGraw et al., 2018; Tournier et al., 2018). Around the world, BDZ are among the most widely prescribed psychotropic drugs, being extensively recommended as anxiolytic, anti-depressive agents, and muscle relaxants: not only available estimates suggest prevalence rates ranging from 9 to 13% of all adult population both in the United States and in Europe (Morgan et al., 1988; Ohayon and Lader, 2002; Ilyas and Moncrieff, 2012; Manthey et al., 2014; Bénard-Laribière and Pariente, 2018; Kowalski-McGraw et al., 2018; Tournier et al., 2018), but especially in the elderly population BDZ are often used for extended periods of time, particularly in older age groups, and in Western Europe (Food Drug Admimistration, 1980; Morgan et al., 1988; Ohayon and Lader, 2002; Lechevallier-Michel et al., 2005; de Santé, 2007; Olfson et al., 2015; Specialist Pharmacist in Substance Misuse, 2020).

BDZ are not harmless, whether used long-term, short-term, or as needed. Global CNS inhibition leads to adverse effects via every area of the brain, eliciting a transitory impairment of psychomotor performances. Moreover, alterations in the pharmacokinetics and pharmacodynamics of BDZ, combined with age-related decrease in the reserve of the central nervous system are liable to lead older age subjects to be particularly sensitive to the cognitive side effects of BDZ (Manthey et al., 2014; Kowalski-McGraw et al., 2018). More precisely, BDZ slow the rate of information processing, impairing alertness and attention, visual processes, motor coordination, and memory. Collectively, these deficits may increase the likelihood of being involved in an accident, typically due to falls and motor vehicle collisions, the latter resulting from compromised steering, road positioning and reaction times, particularly for anxiolytic BDZ (Dassanayake et al., 2011; Elvik, 2013). However, while some evidences hint toward significantly increased risk for motor vehicle collisions compared with non-users (Barbone et al., 1998; Smink et al., 2010; Dassanayake et al., 2011; Elvik, 2013), and the use of BDZ has the potential to negatively affect the performance of safety-sensitive tasks at work (e.g., driving and operating machinery), with eventually increased risk for occupational injuries (Guina and Merrill, 2018; Kowalski-McGraw et al., 2018), the role of BDZ in occupational injuries remains controversial. In fact, available evidence for occupational injuries in BDZ use is scant, particularly when compared with that drawn from studies on the contribute of opioids or alcohol on the risk for work-related injuries (López-Arquillos et al., 2017).

This is particularly frustrating because of the current lack of a common framework or established guidelines regarding the use of BDZ in the workplaces (Brcak et al., 2018; Kowalski-McGraw et al., 2018; Nkyekyer et al., 2018), and/or optimum prescribing practice that may minimize their adverse impact on an individual's injury risk. For example, even in countries whose legislation on health and safety at work does include workplace drug testing, BDZ are usually not included in the screening tests (Kazanga et al., 2012; Mura et al., 2012; Rosso et al., 2017). However, as the common European framework requires the employer to ban drugs at work if there is a considerable danger, while the employee should be in a state that does not endanger himself or others, characterizing the actual risk of occupational injuries in BDZ users may significantly contribute to the daily practice of Occupational Physicians (Pierce, 2012).

For the first time, in this systematic review we therefore retrieved all available evidences, estimating the frequency and the risk for occupational injuries in BDZ users by means of a meta-analytic approach.

## Materials and Methods

### Search Strategy

This systematic review has been conducted according to the PRISMA (i.e., *Prepared Items for Systematic Reviews and Meta-Analysis*) guidelines. Two different databases (PubMed, Embase, and Scopus) were inquired for relevant studies published from their inception to April 3, 2021. The search strategy was a combination of the following keywords [free text and Medical Subject Heading (MeSH) terms]: (benzodiazepine^*^ OR ≪psychotropic drug^*^≫ OR sedative^*^ OR hypnotic^*^ OR ≪sleeping pill^*^≫ OR ≪sleeping tablet^*^≫) AND (accident^*^ OR injur^*^) AND (occupational OR ≪work related≫). Further studies were retrieved from reference lists of relevant articles and consultation with experts in the field. Records we handled using a references management software Endnote X7 software (Thomson Reuters, New York, NY, USA) and duplicates were removed.

### Study Selection

We included all studies, either case-control and cross-sectional, reporting on the use of BDZ in injuries occurring in occupational settings. Cross-sectional studies were included only if a subset of occupational injuries was clearly discernable (i.e., truck or bus drivers, commercial drivers, etc.). To be included, a study should also report the share of injured subjects positive for BDZ use: post-accident analysis of laboratory specimens, questionnaire-based researches, or retrospective assessment of institutional databases were included in the analyses and separately analyzed. Proxies for use of BDZ, i.e., sedatives, sleeping tabs/pills, anxiolytics, were similarly included in the analyses, while more generic case definition (i.e., use of psychotropic drugs) were excluded. In doubtful cases, i.e., the actual shares of positivity for BDZ or their proxies were not overtly reported, corresponding author of the paper was contacted requesting for clarifications. We further explored the reference lists of recent topic-specific reviews to find additional eligible papers. Only articles in English, French, German and Italian were included.

### Data Extraction

Identified studies were independently reviewed for eligibility by two authors (FB and GG) in a two-step-based process: a first screen was performed based on titles and abstracts while full texts were retrieved for the second screen. At both stages, disagreements by reviewers were resolved by consensus.

At the second screen level, data were extracted by one author (GG) supervised by a second author (MR) using a standardized data extraction spreadsheet. The data extraction spreadsheet was piloted on 3 randomly selected papers, and modified accordingly with. Data extraction included: study information (author, year, country), study design (i.e., case control vs. cross-sectional), characteristics of the study group(s), assessment of BDZ positivity status (i.e., laboratory analyses, questionnaire, retrospective assessment of institutional database), total number of participants, population size, and reported estimates (prevalence, OR) or the information needed to calculate an estimate.

Quality of the studies included in meta-analysis was assessed through the Guide methodology for systematic review. Two authors (GG, SG) independently assessed the following domains: recruitment strategy, blinding, exposure assessment, outcome assessment, confounding, incomplete outcome data, selective reporting, and conflict of interest. The quality of individual studies was rated based on fixed and unequivocal criteria in which the end result is one of the following possible statements about the risk of bias: “low,” “probably low,” “probably high,” “high.” Disagreements among raters were resolved through discussion so that a consensus was obtained.

### Data Availability Statement

The data that support the findings of this study are available on request from the corresponding author. The data are not publicly available due to privacy restrictions.

### Statistical Analysis

We first performed a descriptive analysis to report the characteristics of the included studies. The prevalence, OR and corresponding 95% Confidential Intervals (CI) were used as the primary measures to assess the frequency and risk of a positive status for the use of BDZ (or their proxies) in occupational injuries. The results were considered statistically significant when *p* < 0.05.

*I*^2^ statistic was calculated to quantify the amount of inconsistency between studies; it estimates the percentage of total variation across studies that is due to heterogeneity rather than chance. *I*^2^ values ranging from 0 to 25% were considered to represent low heterogeneity, from 26 to 50% as moderate heterogeneity and above 50% as substantial heterogeneity, being pooled using a random-effects model.

To investigate publication bias, contour-enhanced funnel plots representing Egger test for quantitative publication bias analysis (at a 5% of significance level) were generated. In case of asymmetry at the funnel plots, outliers were excluded irrespective of the results of Egger's test. In fact, Egger's test may yield false positive results if fewer than 10 studied were included. Radial plots were then calculated and visually inspected to rule out small study bias.

All calculations and illustrations of funnel plots, were performed by means of “*meta*” and “*metafor*” packages with R (version 4.0.3) and RStudio (version 1.1.463) software.

## Results

### Identification of Studies

**Figure 1:**
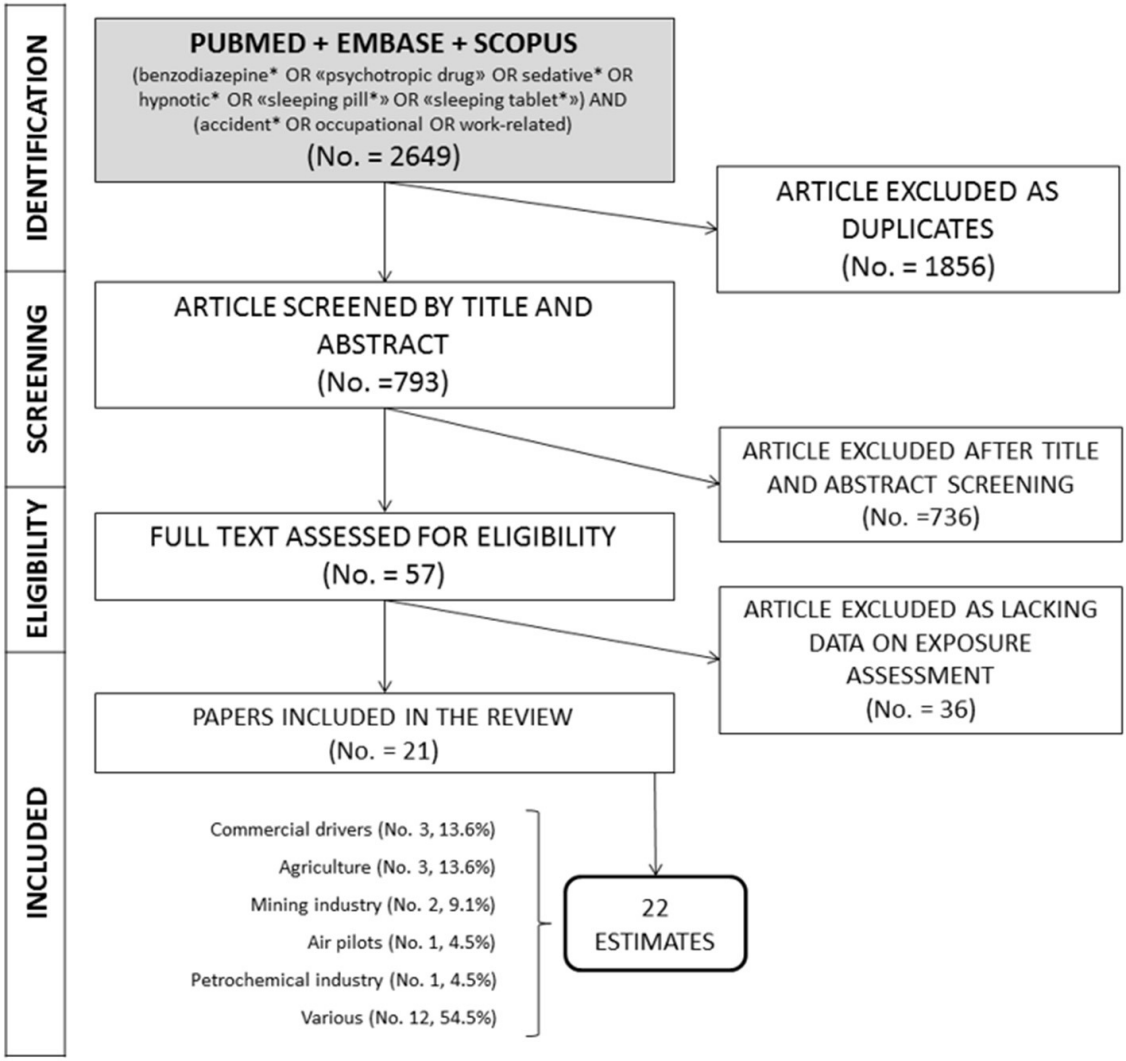
Preferred Reporting Items for Systematic reviews and Meta-Analysis (PRISMA) flow diagram for inclusion of studies in the meta-analysis.

The search strategy yielded a total of 2,649 records (Figure 1). After removing duplicates, 793 abstracts were screened, and 57 full text were assessed for eligibility by titles and abstracts. Of them, a total of 21 full-text articles were screened for their content (Annex 1 in Supplementary Material), and eight further studies were excluded as based on a proxy for BDZ consumption rather than on report about BDZ or their metabolites. Eventually, 13 articles met all the inclusion criteria and were included in the review. All the selected full-text articles reported information about prevalence of BDZ positivity in occupational injuries, and thus have been included in the prevalence's meta-analysis and for the evaluation of the Odds Ratio (OR), to define the risk of injuries BDZ users.

### Studies Characteristics

The pooled characteristics of the included studies are reported in the Table 1. Among the 13 studies, one included two distinctive occupational groups (Szwarc et al., 2009), with a total of 22 estimates. Focusing on the study design, four estimates (28.6%) were based on case control studies (Montastruc and Charlet, 1992; Price, 2012, 2014; Palmer et al., 2014), while remaining estimates were from cross-sectional studies (71.4%) (Girre et al., 1988; Currie et al., 1995; Trucco et al., 1998; Drummer et al., 2003; Kurzthaler et al., 2005; Szwarc et al., 2009; Orriols et al., 2011; Canfield et al., 2012; Nkyekyer et al., 2018). The majority of the estimates were from France (35.7%) and United States (28.6%), with two studies form the United Kingdom (14.3%), a one study from Austria, Australia, Chile (7.1% each one). Among the sampled studies, nine estimates (64.3%) included a mixed sample of occupational injuries referring to specific laboratories and/or occupational medicine services; three further estimates included commercial drivers (21.4), while one sample included fatal cases among air pilots, and injuries in the mining industry (7.1%). As two studies on road accidents specifically included data on injured people who were driving a commercial vehicle at the time of the event, such data were also retrieved and collected (Drummer et al., 2003; Orriols et al., 2011).

**Table 1:** Summary of papers included in the analysis.

Papers included in the analysis	13
Estimates included in the analysis	14
Year range	1988–2018
Study design (No./estimates, %)	
Case control	4, 28.6%
Cross sectional	10, 71.4%
Pooled population (No.)	408, 176
Occupational injuries (No./Total, %)	324, 168, 79.4%
Occupational settings (No./estimates, %)	
Commercial drivers	3, 21.4%
Mining industry	1, 7.1%
Air pilots	1, 7.1%
Other (various)	9, 64.3%
Reporting countries (No./estimates, %)	
Austria	1, 7.1%
Australia	1, 7.1%
Chile	1, 7.1%
China	1, 7.1%
France	5, 35.7%
United Kingdom	2, 14.3%
United States	4, 28.6%
Exposure assessment (No./estimates, %)	
Laboratory exams	10, 71.4%
Retrospective analysis of institutional database	3, 21.4%
Questionnaire	1, 7.1%

The final sample included a data on a total of 408,176 subjects, with 324,168 occupational injuries. Of them, 9,205 cases (2.8%) were positive for the use of, resulting from laboratory analyses (10 out of 14 estimates, 71.4%), a retrospective analysis of institutional databases (3/14, 21.4%), with a further study based on a questionnaire assessment (7.1%).

The quality of the included studies is summarized in Table 2. Six studies had a probably high risk of bias for recruitment strategy, as: the injuries information was drawn from institutional databases of healthcare providers or health clinics (Palmer et al., 2014); retrieved data included compensation claims, being potentially inflated by compensation claims associated with non-traumatic disorders (Currie et al., 1995; Orriols et al., 2011; Price, 2012; Nkyekyer et al., 2018); either study population or controls were collected by convenience (i.e., consecutive cases collected by occupational medicine clinics) (Montastruc and Charlet, 1992; Orriols et al., 2011). Furthermore, four studies had a probably high risk of bias in exposure assessment due to the self-reported use of BDZ or because of the retrieval of prescriptions from institutional databases (Montastruc and Charlet, 1992; Orriols et al., 2011; Palmer et al., 2014; Nkyekyer et al., 2018).

**Table 2:** Assessment of quality of the studies included in meta-analysis: review authors' rates about each domain of risk of bias for each included study (L, Low risk; PL, Probably low risk; PH, Probably high risk; H, High risk).

	**Domains of risk of bias**							
**Studies**	**Recruitment strategy**	**Exposure assessment**	**Blind status**	**Outcome assessment**	**Confounding**	**Incomplete outcome data**	**Selective reporting**	**Conflict of interest**
**Laboratory based**								
Girre et al. (1988)	PL	PL	PH	PL	PH	L	L	PL
Currie et al. (1995)	PH	PL	PH	PL	PH	L	L	PL
Trucco et al. (1998)	PL	PL	PH	PL	PH	PH	PH	PL
Drummer et al. (2003)	PL	L	PH	PH	PL	L	L	L
Kurzthaler et al. (2004)	L	PL	PH	L	PL	L	L	L
Szwarc et al. (2009)	L	PL	PH	L	PH	L	PL	L
Price (2012)	PH	PL	PH	L	L	L	PL	PH
Price (2014)	L	PL	PH	L	L	L	PL	PH
Canfield et al. (2012)	PH	L	PH	L	L	L	PH	PL
**Questionnaire based**								
Montastruc and Charlet (1992)	PH	PH	PH	PL	PH	PH	PH	PH
**Institutional database**								
Orriols et al. (2011)	PH	PH	PH	PH	L	PL	PL	PL
Palmer et al. (2014)	PL	PH	PL	L	PL	L	PL	PL
Nkyekyer et al. (2018)	PH	PH	PL	PH	H	H	PL	PH

In the majority of studies, blind status of researchers may have been high or very high, but because of the retrospective design its eventual effect was presumptively not significant. Most of studies had a probably low risk of bias in the outcome assessment, as employed institutional or administrative data, that are assumed to have a high degree of completeness. Confounding was identified at high risk of bias in the majority of studies, because multiple important potential confounders were not evaluated. Three studies had a high or probably high risk of bias in incomplete outcome data as there was insufficient evidence that such data were adequately addressed. We assigned a low or probably low risk of bias in selective reporting to the majority of studies, while it was significantly higher in non-externally validated questionnaire studies (Montastruc and Charlet, 1992; Canfield et al., 2012). Most studies had a low or probably low risk of bias in conflict of interest, that cannot be ruled out for studies performed by personnel of occupational medicine clinics and/or healthcare providers (Montastruc and Charlet, 1992; Price, 2014).

The main characteristics of the studies we included in the analyses are reported in Annex 1 in Supplementary Material.

### Occurrence of BDZ Use Among Injured Workers

As shown in Table 3, we first explored the raw frequency of BDZ use among injured workers, in all the studies as a whole and by subgroups. The frequency of positivity was 9,205 cases out of 324,168 injuries cases, i.e., 2.71% (95% CI: 1.45–4.98). The majority of index cases were from cases-crossover studies (98% of total occupational injuries), from retrospective analysis of institutional databases (98.2%), and were from a mixed occupational setting (99.6%). Interestingly enough, the estimate prevalence was lower for studies based on institutional databases (1.25%, 95% CI 0.24–6.30), followed by laboratory analyses (3.10%, 95% CI 1.68–5.65), and the only questionnaire questionnaire-based study we eventually processed (8.84%, 95% CI 6.21–12.43). Assuming the laboratory analyses as a reference category, a Risk Ratio (RR) for the positive status regarding BDZ use was 0.927 (0.783–1.097) for cases retrieved through an institutional database, and 3.362 (2.286–4.945) in questionnaire-based estimates. Focusing on the occupational settings, commercial drivers were characterized by a lower share of BDZ positivity (0.73%, 95% CI 0.12–4.30), followed by air pilots (1.55%, 1.01–2.37), and mining industry (2.22%, 0.56–8.45), while studies based on various settings had the higher occurrence (4.28%, 2.47–7.32%). In facts, assuming the latter group as the reference one, the risk of identifying a positive status for BDZ use was considerably lower in commercial driver (RR 0.109, 95% CI 0.063, 0.187) and air pilots (RR 0.539, 95% CI 0.352–0.825). Given the high heterogeneity of the results (*I*^2^ = 94.8% for the sample as a whole, with *I*^2^ > 90% for all subgroups), a random effect model was applied in further analyses.

**Table 3:** Meta-analyses of selected studies by estimates types, exposure assessment, and occupational settings (95% CI = 95% Confidence Interval).

	**No. of estimates**	**Occupational injuries (No./324,168, %)**	**Positive cases (No./9,205, %)**	**Risk ratio (95% CI)**	**Pooled estimated prevalence (No./100 injuries, 95% CI)**	**Chi squared *p*-value**	**Q statistic (df, tau^**2**^)**	***I*^**2**^ (%)**
**Study type**						0.528		
Case control	4	3,844, 2.3%	102, 2.0%	REF	2.85 (1.11; 7.11)		178.46 (9, 1.582)	95.0%
Case-crossover	10	320,324, 97.7%	9,103, 98.0%	0.934 (0.770; 1.132)	2.65 (1.20; 5.73)		63.44 (3, 0.833)	95.3%
**Exposure assessment**						<0.001		
Laboratory	10	5,057, 1.6%	133, 1.4%	REF	3.10 (1.68; 5.65)		133.88 (9, 0.847)	93.3%
Institutional database	3	318,783, 98.3%	9,043, 98.2%	0.927 (0.783; 1.097)	1.25 (0.24; 6.30)		58.37 (2, 2.117)	96.6%
Questionnaire	1	328, 0.1%	29, 0.3%	3.362 (2.286; 4.945)	8.84 (6.21; 12.43)		-	-
**Occupational settings**						<0.001		
Commercial drivers	3	4,151, 1.3%	13, 0.1%	0.109 (0.063; 0.187)	0.73 (0.12; 4.30)		36.40 (2, 2.144)	94.5%
Air pilots	1	1,353, 0.4%	21, 0.2%	0.539 (0.352; 0.825)	1.55 (1.01; 2.37)		-	-
Mining industry	1	90, <0.1%	2, <0.1%	0.772 (0.196; 3.040)	2.22 (0.56; 8.45)		-	-
Other (various)	9	318,574, 98.3%	9,169, 99.6%	REF	4.28 (2.47; 7.32)		184.38 (8, 0.690)	95.7%
**All estimates**	14	324,168, 100%	9,205, 100%		2.71 (1.45; 4.98)		249.26 (13, 1.315)	94.8%

### Risk of Occupational Injuries and Benzodiazepines

**Figure 2:**
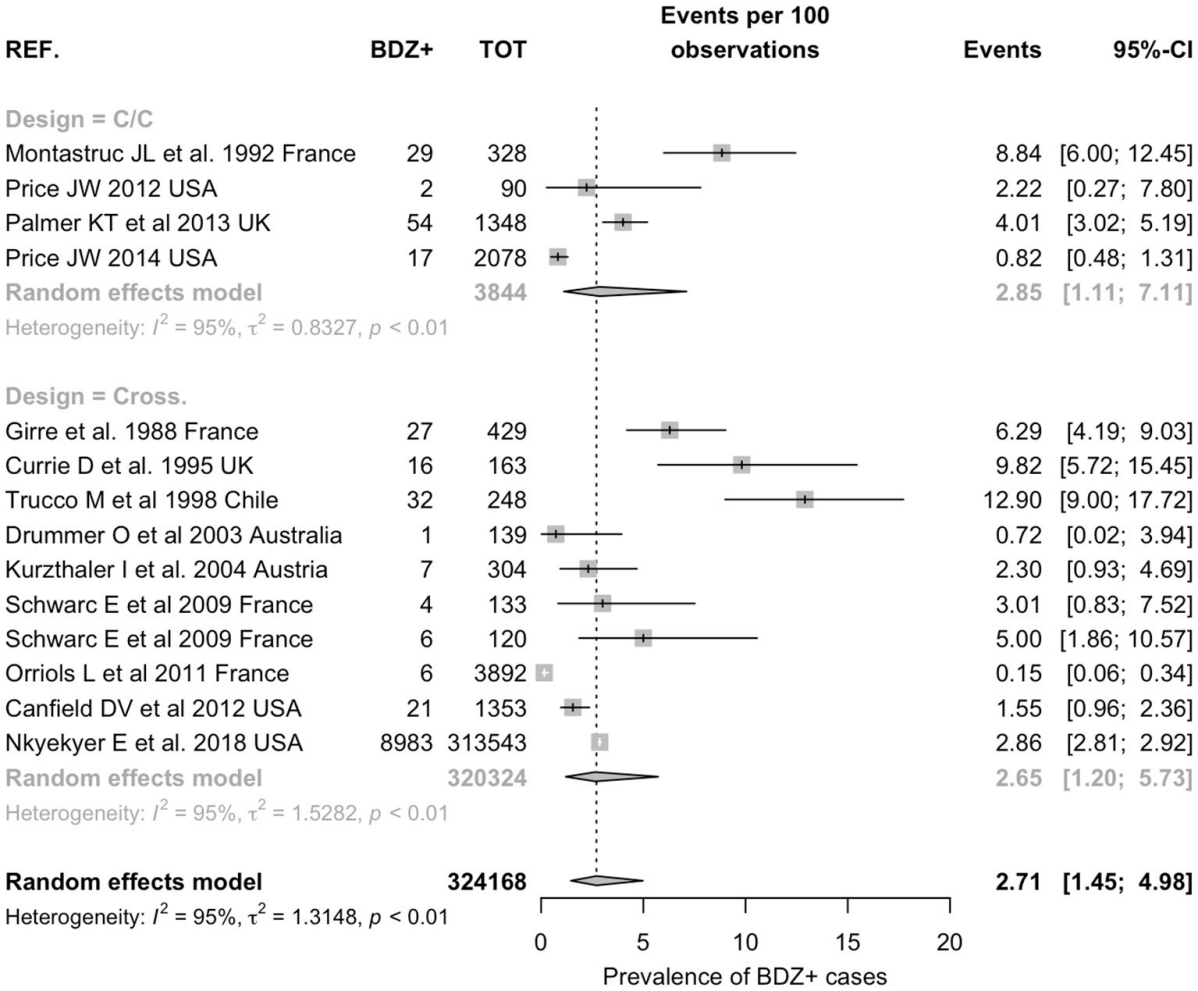
Prevalence of positive status for benzodiazepine use in occupational injuries. Pooled prevalence was estimated in 2.71% (95% Confidence Interval 1.45, 4.98) with substantial heterogeneity (95%).

**Figure 3:**
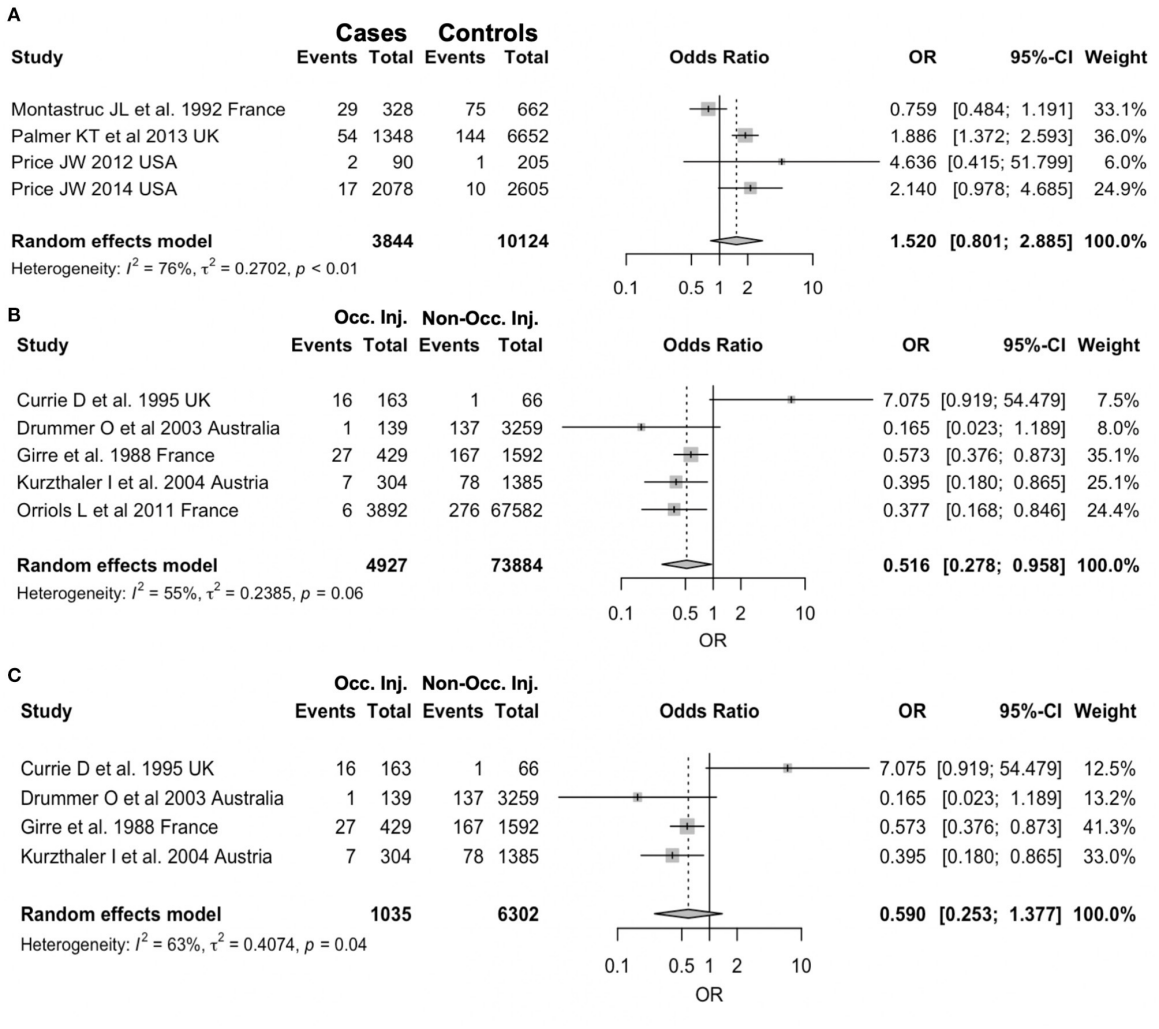
Forest plots for study specific Odds Ratios (OR) with their correspondent 95% confidence intervals (CI) stratified by study design. **(A)** Case-Control studies: positive cases among injured workers vs. positive controls from general population. **(B)** Cross sectional studies: positive cases in occupational injuries vs. non-occupational injuries. **(C)** Cross sectional studies: only laboratory-based studies.

Prevalence of positive status for benzodiazepines use in occupational injuries was estimated in 2.71% (95% CI 1.45–4.98) with substantial heterogeneity (94.8%, *p* < 0.01; Figure 2). No significantly increased referral of BDZ use was reported in occupational injuries when compared with controls from the general population (OR 1.520, 95% CI 0.801–2.885), with substantial heterogeneity across studies (*I*^2^ = 76%, *p* < 0.01; Figure 3A). Regarding cross-sectional studies, a significantly reduced frequency of BDZ use was identified in injured workers when compared with non-injured workers (OR 0.516, 95% CI 0.278–0.958), with a still substantial heterogeneity (*I*^2^ = 55%, *p* = 0.06) (Figure 3B), but the difference substantially disappeared with the analyses were focused on laboratory-based studies (OR 0.590, 95% CI 0.253–1.377), despite substantial heterogeneity (*I*^2^ = 63%, *p* = 0.04) (Figure 3C).

### Publication Bias

**Figure 4:**
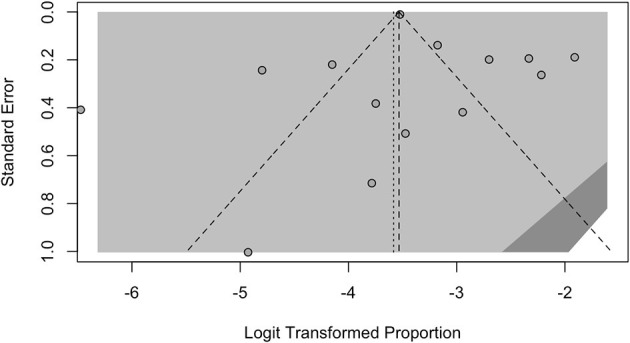
Border-enhanced funnel plots for all studies included in the meta-analysis.

**Figure 5:**
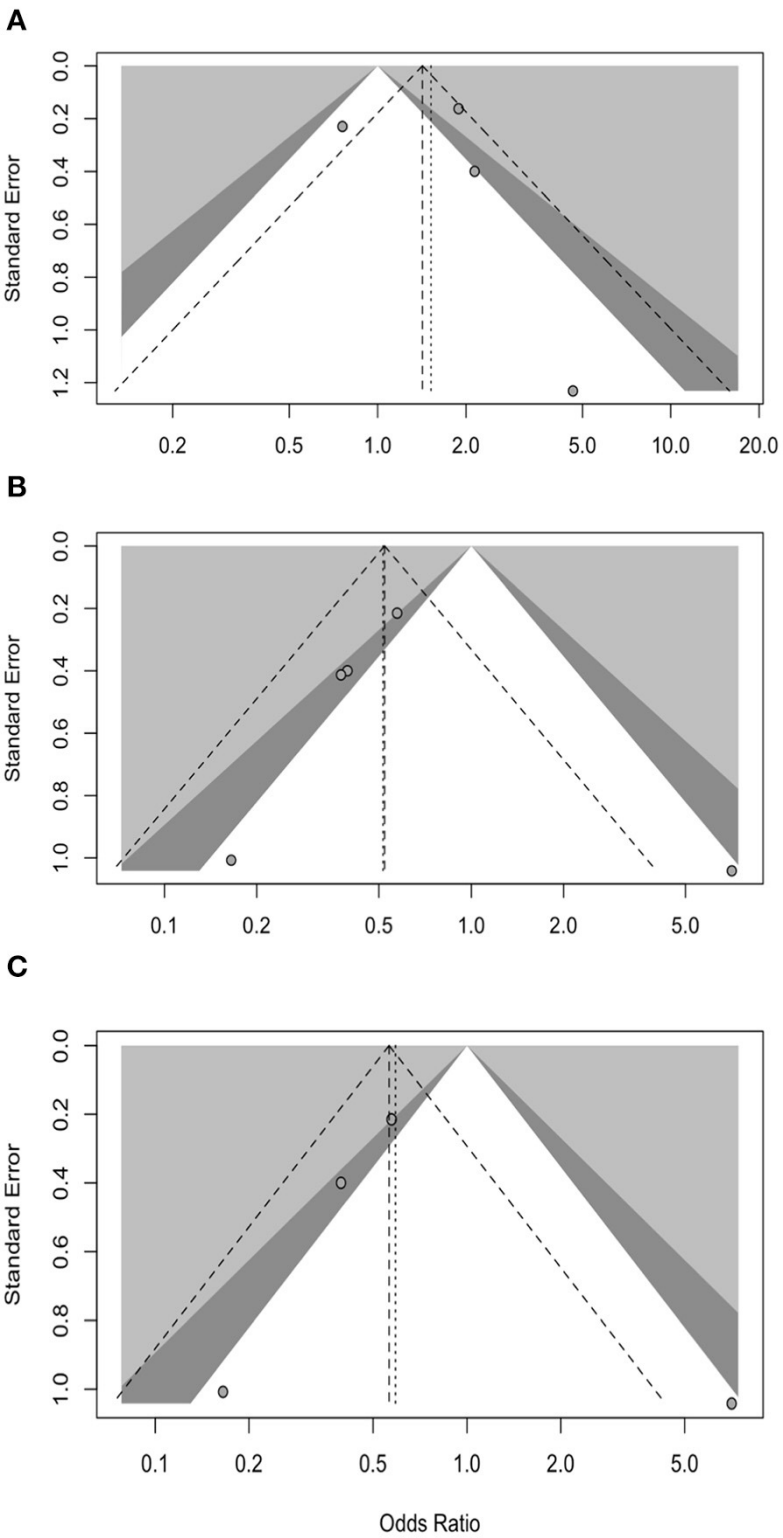
Border-enhanced funnel plots for studies included in the meta-analysis: **(A)** case-control studies comparing positive cases among injured workers vs. positive controls from general population; **(B)** cross-sectional studies comparing positive cases in occupational injuries vs. non-occupational injuries; **(C)** cross-sectional studies comparing positive cases in occupational injuries vs. non-occupational injuries including only laboratory-based estimates.

Visual inspection of contour-enhanced funnel plots showed no substantial evidence of publication bias; this was quantitatively validated by Egger test, not only for the whole of the retrieved studies (*p* = 0.5802) (Figure 4; Annex 2 in Supplementary Material), but also for the subgroup analyses (Figure 5; all analyses *p* > 0.05, Annex 2 in Supplementary Material). As visual inspection of the corresponding radial plots (Annex 2 in Supplementary Material) suggested that the sampled studies randomly scattered around the regression line. This behavior supports that our estimates are not affected by small-study bias.

## Discussion

A growing base of evidence links pre-injury BDZ use with both road accidents and falls of inpatients (Dassanayake et al., 2011). On the contrary, the association between their intake and occupational injuries still remains more doubtful. This is the first metanalysis that evaluated the occupational risk of injuries induced by BDZ. Twenty full-text articles met all the inclusion criteria and were included in the prevalence's meta-analysis and for the evaluation of the OR. The final sample included a total of 324,168, occupational injuries: 2.71% (1.45–4.98) were positive for the use of BDZ resulting from laboratory analyses (10 out of 14 estimates, 71.4%), retrospective analysis of institutional databases (3/14, 21.4%), questionnaire assessment (1/14 estimates, 7.1%). When we focused our analyses only on studies that reported the actual use of BDZ, removing from our estimates all studies based on proxies for use of BDZ (i.e., use of psychoactive drugs, use or “sleeping pills,” etc), available evidence did not suggest any increased risk for occupational among injuries BDZ users. However, the substantial heterogeneity we identified for both general estimates (*I*^2^ 95%, see Figure 2) and for most of the subgroup analyses we performed (all *I*^2^ estimates >55%) precludes drawing more definite conclusions.

Interestingly, the estimate prevalence was lower for studies based on laboratory analyses (3.10%, 95% CI 1.68–5.65), and higher in questionnaire-based studies (8.84%, 95% CI 6.21–12.43), but some explanations may be tentatively suggested. Firstly, laboratory-based studies assess the potential effect of BDZ at the time of the event, hinting toward the actual effects of the drugs on the CNS, while retrospective ones (i.e., database or questionnaire-based) more appropriately evaluate chronic consumption of the drugs. In other words, not only retrospective analyses are more likely to report on baseline neuropsychological disorders of the workers than on their status immediately before the accident. Moreover, our analysis was able to retrieve only one questionnaire-based study (Montastruc and Charlet, 1992), with obvious consequences on the reliability of resulting estimates.

This relative paucity of studies focusing on the occupational consequences of common occurrence (i.e., the use of BDZ) may be explained through the actual requirements for BDZ detection. While alcohol, and even some illicit drugs such as tetrahydrocannabinoids and opioids may be detected on site, either through breathalyzer or urine testing, BDZ detection still requires analysis by a toxicology lab, which involves time and expense as well as sophisticated instruments operated by highly qualified staff (Barbone et al., 1998; Drummer et al., 2003; Kurzthaler et al., 2005; Orriols et al., 2011; Canfield et al., 2012; Brubacher et al., 2016; López-Arquillos et al., 2017; Guina and Merrill, 2018; Nkyekyer et al., 2018). Second, it should be stressed that—being the uptake of BDZ in the general population quite common, laboratory-based studies are not exempt from potential overestimates. In facts, without taking into account the different pharmacokinetic characteristics as the plasma half-life, and the presence of an active metabolite of BDZ, even laboratory-based studies may report positive status that are in fact devoid of any functional and/or biological impact on neurological functions. In this regard, only three articles addressed the different pharmacokinetic characteristics as the plasma half-life and the presence of an active metabolite of BDZ or BDZ-like hypnotics (Bramness et al., 2002; Kurzthaler et al., 2004; Orriols et al., 2011; Price, 2012, 2014).

In order to overcome such limitation, many studies have rather assessed some proxies for BDZ use, either focusing on a more general statement and/or requirements (i.e., use of psychotropic drugs, that therefore include also BDZ, etc.), or through the analysis of institutional databases for previous receipts, or even questionnaires (Drummer et al., 2003; Brubacher et al., 2016), but such approaches necessarily elicit significant approximations. Firstly, by asking the injured worker on the use of BDZ through a questionnaire retrieving “*sleeping pills*” or “*sleeping tablets*” or “*drugs for anxiety*” or “*drugs for insomnia*,” the eventual estimates may improperly include more intrusive drugs, such as Z-drugs, or neuroleptics (Bramness et al., 2002; Orriols et al., 2011; Price, 2012, 2014; Nkyekyer et al., 2018). Moreover, as the impairment of neurological functions following the uptake of such medications has a clear concentration-effect relationship, as for drivers, any retrospective inquiry on the use of BDZ and related drugs may be either inflated or overlooked because of retrieving bias of social desirability bias (Bramness et al., 2002). In facts, a limitation of most of estimates, either database- or questionnaire-based, but also shared by most of laboratory-based studies, is the lack of data about the actual posology and the settings of drug uptake, in acute and/or chronic form, impairing an appropriate definition of the time limit (Movig et al., 2004; Herrera-Gómez et al., 2018).

Even though such variables as the posology and the settings of drug uptake, in acute and/or chronic form, strictly model the impact of BDZ on the cognitive side and on the psychophysical performances, only two studies have taken in account such factors in their analyses (Orriols et al., 2011; Nkyekyer et al., 2018). This is particularly important as long-term use of BDZ carries anterograde amnesia, depressive symptoms and suicidal ideation and the risk of cognitive impairment (Paterniti et al., 2002; Barker et al., 2004), even without brain abnormalities visible on neuro-imaging (Busto et al., 2000). The effect may be due to a permanent depletion of cognitive reserve (Stern, 2002; Sterm, 2006; Smink et al., 2010), with possible but strikingly heterogeneous differences in resulting cognitive function even in individuals with identical levels of neuropathology (Stern, 2002, 2012). If other words, if BDZ actually deplete cognitive reserve, then a lower level of neuropathology would be sufficient to reach the diagnostic threshold for cognitive impairment and reduced of psychomotor performance (Penninkilampi and Eslick, 2018). As a consequence, to correctly interpret the effect on the cognitive side of long-term BDZ use, it would be important to know the motivation of taking them and the neuropsychological condition of the worker in order to distinguish preclinical conditions. The question is still highly debated today. Indeed, in two large sample size studies the highest lifetime cumulative use of BDZ was not associated with a higher risk of dementia than any use (Imfeld et al., 2015; Biétry et al., 2017).

An interesting remark of our study, is the surprisingly low risk for occupational injuries in professional drivers. This is particularly interesting as the use of BDZ has been reported to increase crash risk up to 100 times (Movig et al., 2004). This is particularly interesting as the use of BDZ appears less frequent among professional drivers (10.97%) than in the general populational (15.38%) (Herrera-Gómez et al., 2018), particularly when dealing with the daily use (i.e., 2% in the population vs. 1% of professional drivers took these drugs every day). A possible explanation may be found both in practical issues and in the legal framework outside of the occupational settings. On the one hand, commercial and professional drivers are more frequently requesting for drugs countering sleep and rest than those against anxiety and insomnia (Drummer et al., 2003; Movig et al., 2004; Herrera-Gómez et al., 2018). On the other hand, many high-income countries have developed various legal mechanisms to address drugs and driving,^1^ either separating or combining the objectives of road safety and control of illicit drugs. However, a few countries refer to a list of substances that drivers may not use: BDZ are not consistently reported in such lists, as well as for some psychoactive substances such as medicines or new psychoactive substances which are not yet under control.^1^ As a consequence, commercial drivers may deliberately avoid the use of BDZ, and this behavior may elicit a paradoxical effect: being the commercial drivers often affected by work-related stress, and by poor sleep quality, they could benefit from an appropriate (certainly not “homemade”) use of BDZ.

In conclusion, the effect of the short or long-term use of BDZs on psychomotor performance and cognitive aspects is still unclear as the data are still discordant due to the lack of rigor in the collection of information on drug intake in the studies. It would be relevant to have information on the type of BDZ taken, dosage, time of intake, indication of the prescription of BDZ and concomitant use of other drugs, temporal correlation with the possible injury.

Certainly the long-term use of BDZs, especially if they have a long half-life, have deleterious effects on performance and cognitive aspects with increased risk of injuries, the effect size for the use of short-acting BDZ being smaller than for long-acting BDZ.

The use of short-acting BDZ for limited periods, as is currently the optimum prescribing practice, may minimize the adverse impact on an individual's cognitive impairment and in depressive patients it could improve the information processing speed after acute treatment (Duan et al., 2019).

It is imperative that greater efforts are made to curtail inappropriate prescribing, keeping BDZ for selected circumstances only preferring short-acting BDZ for limited periods. This is important not only for the long-term prevention of cognitive impairment or dementia and dependence in a vulnerable population but also to decrease the risk of incidence and injury in workplace. Since BDZs are among the most used drugs in the world with a high risk of creating addiction and data in the literature suggest that the use of hypnotics represent an avoidable risk factor with respect to road accidents, more work is needed to better define the association between use of BDZ and occupational injury risk and subsequent morbidity.

## Data Availability Statement

The original contributions presented in the study are included in the article/Supplementary Material, further inquiries can be directed to the corresponding author.

## Author Contributions

SG and MR: conceptualization. MR, NB, SG, and PL: methodology. MR, NB, and GG: formal analysis. SG and PL: data analysis. SG, PL, MR, NB, and GG: data curation, writing—review and editing, and writing—original draft preparation. All authors have read and agreed to the published version of the manuscript.

## Conflict of Interest

The authors declare that the research was conducted in the absence of any commercial or financial relationships that could be construed as a potential conflict of interest.

## Footnotes

^1^https://www.emcdda.europa.eu/publications/topic-overviews/legal-approaches-to-drugs-and-driving/html_en# (visited March 2021).

## Supplementary Material

Annex 1 Studies selected conducting the search strategy in PubMed, EMBASE and SCOPUS. Retrieved studies are reported in chronological order.

Annex 2 Radial plot for small study bias. The radial plots display point estimates (y-axis) with different standard errors (x-axis). According to Galbraith, 1990 there is no small-study effects, individual studies are expected to scatter randomly around the regression line through the origin. Radial plots were specifically calculated for the whole of data **(a)**; for the case-control studies **(b)**; cross sectional studies comparing positive cases among injured workers vs. positive cases among non-injured workers **(c)** and positive cases in occupational injuries vs. non-occupational injuries **(d)**, and cross sectional studies including only laboratory-based studies **(e)**. In all cases, estimated were substantially scattered on the both sides of the regression lines, suggesting no significant small study effect.
