# Supplementary material for: Occupational Injuries and Use of Benzodiazepines: A Systematic Review and Metanalysis
Source: Front Hum Neurosci. 2021 May 13;15:629719. doi: 10.3389/fnhum.2021.629719 (PMC8155305; doi:10.3389/fnhum.2021.629719)
Supplement: Annex 1 — Studies selected conducting the search strategy in PubMed, EMBASE and SCOPUS. Retrieved studies are reported in chronological order. [file Data_Sheet_1.docx]

**ANNEX 1.** Studies selected conducting the search strategy in PubMed, EMBASE and SCOPUS. Retrieved studies are reported in chronological order.

| **Reference, year** | **Study –Type** | **Country** | **Period of study** | **Study populations (sample number)** | **Exposure assessment** | **Drug assessed** | **Main findings** |
| --- | --- | --- | --- | --- | --- | --- | --- |
| **Girre et al., 1988** [30] | *Cross.* | *France* | 1982-1983 | Subjects admitted to the emergency departments of general hospitals (4796) | *Laboratory analyses* | *Benzodiazepine(s)* | 9.6% of subjects in whom the presence of benzodiazepines was detected in the plasma of accident victims.  The mean age of the benzodiazepine consumers was higher, as 23% of the BZD + subjects were over the age of 50 years. |
| **Montastruc et al., 1992** [26] | *C/C* | *France* | 1989 - 1990 | Workers of metropolitan area (990) | *Questionnaire* | *Benzodiazepine(s)* | Comparing BZD consumption by injured people (cases) versus  a non-injured control group (controls) showed no association of BDZ consumption with greater risk of industrial injuries. |
| **Currie et al., 1995** [31] | *Cross.* | *UK* | May to September 1992 | Subjects admitted to the emergency departments with accident injuries sufficiently serious to require the taking of blood (229) | *Laboratory analyses* | *Benzodiazepine(s)* | Greater representation of BDZ in blood taken from a group responsible for an accident as opposed to a group not responsible for an accident |
| **Trucco et al., 1998** [32] | *Cross.* | *Chile* | **//** | Patients admitted to a hospital within 6 hours of an accident severe enough to require hospitalization (238) | *Laboratory analyses* | *Benzodiazepine(s)* | Recent use of alcohol and drugs is frequent among severe work related accident victims |
| **Drummer et al., 2003** [33] | *Cross.* | *Australia* | 1990 to 1999 | Drivers killed in motor vehicle accidents in three Australian states (3398); only injuries occurring in truck/van drivers (i.e. presumptively work related) were retained for analyses (139) | *Laboratory analyses* | *Benzodiazepine(s)* | the use of benzodiazepines was frequently associated to opioid and/or cannabis |
| **Kurtzhaler et al., 2005** [34] | *Cross.* | *Austria* | 1995 | All patients who were admitted within one year to the Department of Traumatology as a result of an accident (1611) | *Laboratory analyses* | *Benzodiazepine(s)* | Benzodiazepine use was also more common in violence related injuries than in any of the other accident categories |
| **Szwarc et al., 2009** [25] | *Cross.* | *France* | 2000-2005 | compiling files on occupational accidents from two different public agencies (500) | *Laboratory analyses* | *Benzodiazepine(s)* | THC (as proven by GC–MS and/or LC–MS/MS) was  present in all 10 victims, together with benzodiazepines in 1 case |
| **Orriols et al., 2011** [35] | *Cross.* | *France* | from July 2005 to May 2008 | extracting and matching data from three French nationwide databases for road traffic accidents (72,685); only cases occurring in truck drivers were retained (3892); | *Institutional database* | *Benzodiazepine(s)* | The risk of being responsible for a traffic accident was higher in users of benzodiazepine hypnotics (odds ratio (or) = 1.39 (1.08–1.79)) and in the 155 drivers to whom a dosage of more than one pill of zolpidem a day had been dispensed during the 5 months before the collision (or = 2.46 (1.70–3.56)). |
| **Price, 2012** [27] | *C/C* | *USA* | June 11, 2009 and October 5, 2011**.** | employees from coal mines: control sample (n = 215) miners that presented for random urine drug testing. study sample (n = 100) miners that presented for postaccident urine drug testing. | *Laboratory analyses* | *Benzodiazepine(s)* | Blood drug testing is a better means of obtaining toxicological evidence of impairment after an accident. (different type of BDZ) |
| **Canfield et al., 2011** [36] | *Cross.* | *USA* | between 2004 and 2008 | pilots who died in aviation accidents (1353) | *Laboratory analyses* | *Benzodiazepine(s)* | **BDZ** drug positives for the past 20 years. Significant aviation performance impairment can be associated with drugs. |
| **Palmer et al., 2014** [28] | *C/C* | *UK* | between 1 January 1987 and 31 December 2009 | anonymised dataset con- taining the full primary care medical records of 9612 cohort members comprising 1602 patients who had consulted their general practice or attended hospital with a qualifying injury code (cases) and 8010 patients with no workplace injury (controls). (9612) | *Institutional database* | *Benzodiazepine(s)* | a range of common mental health illnesses and classes of psychotropic treatment contribute to an important, minority of workplace injury events. |
| **Price, 2014** [29] | *C/C* | *USA* | from January 3, 2008, to June 1, 2013. | employees from various industries (4756) | *Laboratory analyses* | *Benzodiazepine(s)* | The results for comparing the total confirmed laboratory positive benzodiazepine tests controlled for other substances, although suggestive of an association, did not achieve statistical significance. |
| **Nkyekyer et al. 2018** [19] | *Cross.* | *USA* | 2012 – 2015 | State Department of Labor and Industries workers (313,543) | *Institutional database* | *Benzodiazepine(s)* | Pre-injury opioid and benzodiazepine use may increase the risk of disability after work-related injury. |
