# Supplementary material for: Occupational Injuries and Use of Benzodiazepines: A Systematic Review and Metanalysis
Source: Front Hum Neurosci. 2021 May 13;15:629719. doi: 10.3389/fnhum.2021.629719 (PMC8155305; doi:10.3389/fnhum.2021.629719)
Supplement: Annex 2 — Radial plot for small study bias. The radial plots display point estimates (y-axis) with different standard errors (x-axis). According to Galbraith, 1990 there is no small-study effects, individual studies are expected to scatter randomly around the regression line through the origin. Radial plots were specifically calculated for the whole of data (a); for the case-control studies (b); cross sectional studies comparing positive cases among injured workers vs. positive cases among non-injured workers (c) and positive cases in occupational injuries vs. non-occupational injuries (d), and cross sectional studies including only laboratory-based studies (e). In all cases, estimated were substantially scattered on the both sides of the regression lines, suggesting no significant small study effect. [file Data_Sheet_2.docx]

**ANNEX 2**. **Radial plot for small study bias.** The radial plots display point estimates (y-axis) with different standard errors (x-axis). According to Galbraith et al 1990, if there is no small-study effects, individual studies are expected to scatter randomly around the regression line through the origin. Radial plots were specifically calculated for the whole of data (**Panel a**); for the case-control studies (**Panel b**), and for cross sectional studies (**Panel c**), including only laboratory-based studies (**Panel d**). In all cases, estimated were substantially scattered on the both sides of the regression lines, suggesting no significant small study effect.

| 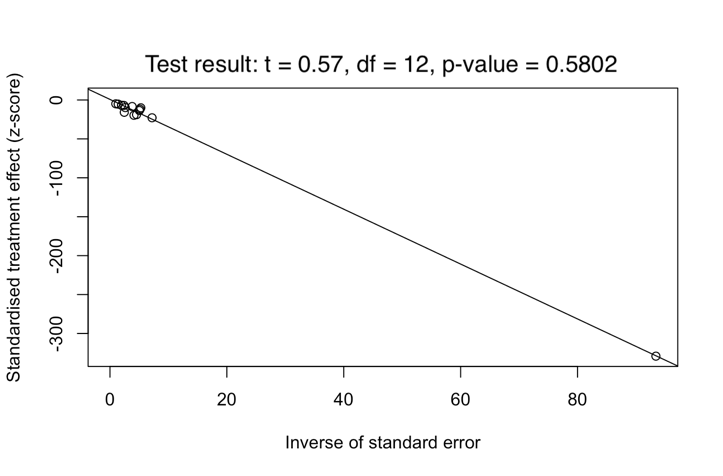 | 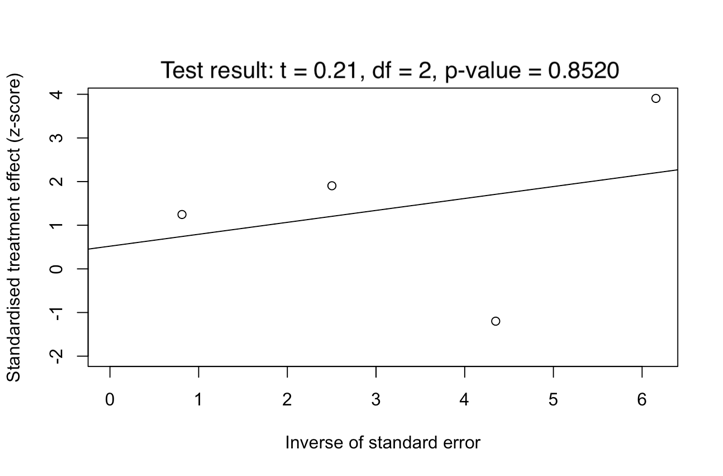 |
| --- | --- |
| a) | b) |
| 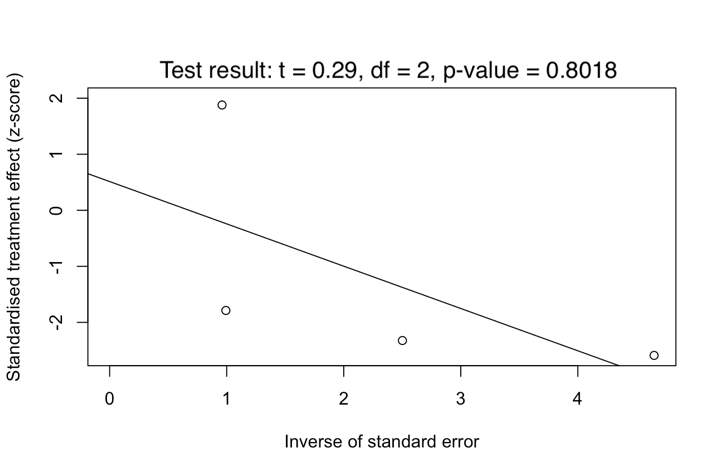 | 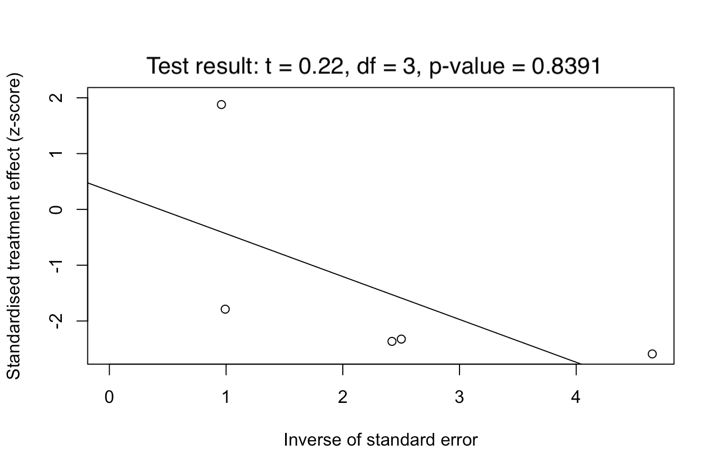 |
| c) | d) |
